# Damage Signaling by Extracellular Nucleotides: A Role for Cyclic Nucleotides in Elevating Cytosolic Free Calcium?

**DOI:** 10.3389/fpls.2021.788514

**Published:** 2021-12-02

**Authors:** Jian Sun, Youzheng Ning, Limin Wang, Katie A. Wilkins, Julia M. Davies

**Affiliations:** ^1^Department of Plant Sciences, University of Cambridge, Cambridge, United Kingdom; ^2^Institute of Integrative Plant Biology, School of Life Science, Jiangsu Normal University, Xuzhou, China

**Keywords:** calcium, CNGC, cyclase, cyclic nucleotide, DAMP, ATP

## Abstract

Extracellular ATP (eATP) is now held to be a constitutive damage-associated molecular pattern (DAMP) that is released by wounding, herbivory or pathogen attack. The concentration of eATP must be tightly regulated as either depletion or overload leads to cell death. In *Arabidopsis thaliana*, sensing of eATP is by two plasma membrane legume-like lectin serine–threonine receptor kinases (P2K1 and P2K2), although other receptors are postulated. The transcriptional response to eATP is dominated by wound- and defense-response genes. Wounding and pathogen attack can involve the cyclic nucleotides cyclic AMP (cAMP) and cyclic GMP (cGMP) which, in common with eATP, can increase cytosolic-free Ca^2+^ as a second messenger. This perspective on DAMP signaling by eATP considers the possibility that the eATP pathway involves production of cyclic nucleotides to promote opening of cyclic nucleotide-gated channels and so elevates cytosolic-free Ca^2+^. *In silico* analysis of P2K1 and P2K2 reveals putative adenylyl and guanylyl kinase sequences that are the hallmarks of “moonlighting” receptors capable of cAMP and cGMP production. Further, an *Arabidopsis* loss of function *cngc* mutant was found to have an impaired increase in cytosolic-free Ca^2+^ in response to eATP. A link between eATP, cyclic nucleotides, and Ca^2+^ signaling therefore appears credible.

## Introduction

Extracellular ATP (eATP) is now recognized as a plant cell regulator, with the ability to affect growth, development, and stress responses (reviewed by Matthus et al., 2019a). With resting levels thought to be in the nanomolar range, eATP concentration must be tightly regulated as either depletion or overload leads to cell death (Chivasa et al., 2005; Sun et al., 2012; Deng et al., 2015). This extracellular nucleotide is now thought to be involved in the response to wounding and microbial attack, such that it is classified as a constitutive damage-associated molecular pattern (DAMP; Choi et al., 2014b; Tanaka and Heil, 2021). It is readily envisaged that a breach of the plasma membrane would allow release of cytosolic ATP to the apoplast. Increased eATP concentration has indeed been measured from *Arabidopsis thaliana* roots, plus *Arabidopsis* and kidney bean (*Phaseolus vulgaris* L) leaves in response to mechanical wounding (Song et al., 2006; Weerasinghe et al., 2009; Dark et al., 2011; Wang et al., 2019a). Wounding can also result in accumulation of extracellular glutamate, which in turn can cause eATP accumulation (Dark et al., 2011; Toyota et al., 2018). Non-damaging mechanical disturbance generated by touch can lead to increased eATP of roots (Weerasinghe et al., 2009), suggesting that microbial growth that does not breach the plasma membrane could cause eATP increase by putting pressure on walls. If the plasma membrane were to remain intact, studies suggest that ATP release mechanisms include exocytosis and a diversity of transport proteins (Kim et al., 2006; Choi et al., 2014b; Matthus et al., 2019a).

Microbial presence has indeed been found to influence eATP levels, which in turn may affect colonization. The early phase of root colonization of both *Arabidopsis* and barley (*Hordeum vulgare*) by the endophytic fungus *Serendipita indica* is associated with significant eATP increase (Nizam et al., 2019). However, secretion of an eATP hydrolyzing ecto-5'-nucleotidase by the fungus lowers eATP and allows greater colonization (Nizam et al., 2019). *Arabidopsis* roots expressing the *S. indica* nucleotidase became more susceptible to colonization by the pathogenic fungus *Colletotrichum incanum*, suggestive of a role for eATP depletion (Nizam et al., 2019). The presence of *Pseudomonas syringae* (and to a lesser extent flg22) results in increased eATP levels in *Arabidopsis* leaf, particularly around guard cells. The high eATP causes stomatal closure, limiting bacterial entry (Chen et al., 2017). The mycotoxin beauvericin (BEA) increases eATP of wheat (*Triticum aestivum*) coleoptiles and causes cell death (Šrobárova et al., 2009). BEA is most notably produced by *Fusarium* species and acts in wheat head blight (Šrobárova et al., 2009). As a Ca^2+^ ionophore (Caloni et al., 2020), BEA could act to increase Ca^2+^ influx into cells. In *Arabidopsis* roots, promoting Ca^2+^ influx results in increased eATP most likely by stimulating exocytotic ATP release (Kim et al., 2006), which may help explain BEA’s ability to elevate wheat eATP. In contrast, the mycotoxin Fumonisin B1 (FB1) causes eATP depletion in *Arabidopsis* leading to cell death (Chivasa et al., 2005). Delineating how cells sense and respond to varying eATP levels is therefore critical to understanding the role of eATP in regulating what may be a continuum between growth, immunity, and death (Choi et al., 2014b).

The first angiosperm eATP receptor was identified through an *Arabidopsis* forward genetic screen as a plasma membrane legume-like lectin serine–threonine receptor kinase, “DOes not Respond to Nucleotides1” (DORN1), now also known as P2K1 (consistent with animal eATP receptor terminology) and formerly known as LecRK1.9 (Gouget et al., 2006; Bouwmeester et al., 2011; Choi et al., 2014a; Balagué et al., 2017). It is thought to form part of the plasma membrane–cell wall continuum (Bouwmeester et al., 2011). Structurally, DORN1/P2K1 differs markedly from the animal eATP receptors, which are eATP-binding Ca^2+^ channels or G-protein-coupled receptors (Choi et al., 2014a,b; Nguyen et al., 2016; Verkhratsky, 2021). DORN1/P2K1 is important for defense against *Botrytis cinerea* (necrotrophic fungus: Tripathi et al., 2018), *Phytophthora brassicae* and *Phytophthora infestans* (biotrophic oomycetes: Gouget et al., 2006; Bouwmeester et al., 2011, 2014), *P. syringae* (hemibiotrophic bacterium: Balagué et al., 2017; Chen et al., 2017), *Rhizoctonia solani* (necrotrophic fungus: Kumar et al., 2020) and *S. indica* (Nizam et al., 2019). A second *Arabidopsis* eATP receptor, P2K2, was identified by complementation of a *p2k1* mutant. P2K2 is also a plasma membrane legume-like lectin receptor kinase which is trans-phosphorylated by P2K1 and is involved in defense against *P. syringae* (Pham et al., 2020). There is now evidence that DORN1/P2K1 and P2K2 are unlikely to be the only *Arabidopsis* eATP receptors (Zhu et al., 2017, 2020; Matthus et al., 2019a; Smith et al., 2021). Nevertheless, activation of DORN1/P2K1 by eATP leads to phosphorylation of mitogen-activated protein kinase 3 (MPK3; Choi et al., 2014a). MPK3 can phosphorylate calmodulin-binding transcription activator 3 (CAMTA3) in flg22 signaling, which results in CAMTA3 breakdown and the release of its repression of defense gene transcription (Jiang et al., 2020). It is not clear whether this also occurs in eATP signaling, but an estimated 99.8% of seedling eATP-responsive transcriptome requires DORN1/P2K1 and the CAM-box CAMTA3-binding target is highly enriched in promoters of eATP-regulated genes (Jewell et al., 2019). The eATP transcriptome is enriched in wound response and defense-related genes, with some also requiring regulation by the ethylene, jasmonate, and salicylic acid pathways (Choi et al., 2014a; Tripathi et al., 2018; Jewell et al., 2019).

While receptor characterization and analyses of receptor mutants have established eATP as a DAMP, the signaling systems downstream of receptors remain less well understood. A transient increase in cytosolic-free Ca^2+^ ([Ca^2+^]_cyt_) as a second messenger is one of the earliest detectable changes downstream of eATP (Demidchik et al., 2003; Choi et al., 2014a; Behera et al., 2018). Mechanical wounding and insect feeding also increase [Ca^2+^]_cyt_ (Hander et al., 2019; Malabarba et al., 2021), suggesting the involvement of eATP. It can be noted in passing that saliva from insect herbivores can contain eATP hydrolyzing enzymes such as ecto-apyrase, suggesting that eATP is an important target to control (Su et al., 2012; Wu et al., 2012). Studies on how eATP increases [Ca^2+^]_cyt_ in *Arabidopsis* have focused on plasma membrane Ca^2+^-permeable channels. Pharmacological block of such channels prevents [Ca^2+^]_cyt_ elevation (Demidchik et al., 2003; Behera et al., 2018) and adversely affects transcription of jasmonate-dependent genes involved in wounding and defense (Tripathi et al., 2018). Patch clamp electrophysiological analyses of root epidermis have revealed eATP- and DORN1/P2K1-dependent Ca^2+^ influx channel conductances that would be competent to elevate [Ca^2+^]_cyt_ (Demidchik et al., 2009; Zhu et al., 2017; Wang et al., 2018, 2019b). eATP-activated Ca^2+^ influx conductances have also been found in tobacco pollen tube and *Vicia faba* guard cell plasma membranes (Wang et al., 2014; Wu et al., 2018). The identity of the underpinning Ca^2+^ channels in all of these cell types remains under investigation. To date, the plasma membrane NADPH oxidase RBOHC (respiratory burst oxidase homologue C; Demidchik et al., 2009) and the heterotrimeric G protein alpha subunit (GPA1; Zhu et al., 2017) are implicated in regulating *Arabidopsis* root eATP-activated channels. Annexin1 (which supports a hydroxyl radical-activated Ca^2+^ channel activity; Laohavisit et al., 2012) is implicated in eATP-induced [Ca^2+^]_cyt_ increase of *Arabidopsis* roots and transcriptional regulation of wound- and defense-response genes, but its mode of action has not been determined (Mohammad-Sidik et al., 2021). It also underpins wounding- and herbivory-induced [Ca^2+^]_cyt_ increases (Malabarba et al., 2021). Annexin4 supports an eATP-induced [Ca^2+^]_cyt_ increase when expressed in *Xenopus* oocytes, but its *in planta* activity has not yet been reported (Ma et al., 2019). Recently, a patch clamp study on *Arabidopsis* pollen grains identified two cyclic nucleotide-gated channel (CNGC) subunits (CNGC2 and CNGC4) as underpinning a plasma membrane eATP-activated Ca^2+^ influx conductance that may be relevant to germination (Wu et al., 2021). The conductance was lost in the *dorn1* loss of function mutants suggesting the channels act downstream of this receptor although it was not checked whether *CNGC2* and *CNGC4* expression were downregulated in *dorn1*. The CNGC2 and CNGC4 channel subunits form a heterotetrameric plasma membrane Ca^2+^ influx channel in flg22 pathogen-associated molecular pattern-triggered immunity (PAMP; Tian et al., 2019), indicating a possible common point in DAMP and PAMP signaling. Furthermore, the results of Wu et al. (2021) imply that the cyclic nucleotides 3',5'-cyclic adenosine monophosphate (cAMP) or 3',5'-cyclic guanosine monophosphate (cGMP) act downstream of eATP and DORN/P2K1. Although eATP has long been known to increase animal cell cAMP (Choi and Kim, 1997; Castro et al., 1998), there appear to be no reports to date of eATP’s having an effect on cAMP or cGMP levels in plants, but these nucleotides can increase [Ca^2+^]_cyt_ through CNGC activation (reviewed by Jarratt-Barnham et al., 2021). This perspective will consider the possibility that cyclic nucleotides and CNGCs act in eATP-DAMP [Ca^2+^]_cyt_ signaling.

## Cyclic Nucleotide Production by Moonlighting Proteins in Response to Pathogens and Wounding

cAMP and cGMP are involved in growth and development, abiotic stress responses, stomatal kinetics, control of photorespiration and photosynthesis, and immunity (reviewed by Jarratt-Barnham et al., 2021). Production of cAMP and cGMP is now held to be by adenylyl cyclase (AC) and guanylyl cyclase (GC) centers embedded in a diverse range of soluble and membrane-integral proteins (Ludidi and Gehring, 2003; Ruzvidzo et al., 2019; Al-Younis et al., 2021; Turek and Irving, 2021). There is clear evidence supporting cytosolic cyclic nucleotide production by a “moonlighting” function of members of the receptor-like kinase (RLK) superfamily, relevant to response to pathogens, where the catalytic center is present in the cytosolic kinase domain (Qi et al., 2010; Kwezi et al., 2011; Turek and Irving, 2021). For example in *Arabidopsis*, LLRAC1 (leucine-rich repeat adenylyl cyclase 1) is implicated in defense against *P. syringe* and the biotrophic fungus *Golovinomyces orontii* (Bianchet et al., 2019). Wall-associated kinase-like (WAKL) proteins in *Arabidopsis*, wheat and *Brassica napus* are implicated in cGMP production and pathogen defense (reviewed by Turek and Irving, 2021). Most recently, expression of rice WAKL21.2 (which has GC activity) in *Arabidopsis* resulted in transcription of SA-related defense genes and conferred resistance to *P. syringae* (Malukani et al., 2020).

In terms of DAMP signaling, wounding was found to result in a rapid, fivefold increase in *Arabidopsis* leaf cAMP and cGMP by van Damme et al. (2014). In this instance, the 2',5' isomers were detected but the mode of production and downstream consequences remain unknown. Larval oral secretions from the moth *Spodoptera littoralis* can increase cAMP content of *Arabidopsis* leaves within 3 min, which may relate to herbivory damage (Kumar et al., 2020). An initial decreased transcription followed by longer-term increased transcription of *Hippeastrum hybridum Guanylyl Cyclase 1* (*HpGC1*, most likely encoding a soluble enzyme) was found in wounded and infected scales (fungal infection by *Peyronellaea curtisii*). Although a causal relationship was not demonstrated, HpGC1 activity may relate to downstream production of cGMP in the challenged scales (Świeżawska et al., 2015). Since then, triphosphate tunnel metalloenzyme 3 (TTM3) from *Brachypodium distachyon* has been found to have both ATP hydrolyzing and AC activities, with upregulation of transcription by wounding (Świeżawska et al., 2019). There remains a significant knowledge gap when it comes to whether eATP as a DAMP (or in its role as growth regulator) has an effect on cAMP or cGMP levels. However, increasing cAMP through expression of an AC increases transcription of wound-related genes (Xu et al., 2021) which could place eATP (as a DAMP governing wound-related transcription) upstream of cAMP production. A key question is whether the eATP receptors could themselves generate cAMP or cGMP.

## Could eATP Receptors Generate cAMP or cGMP?

**Figure 1:**
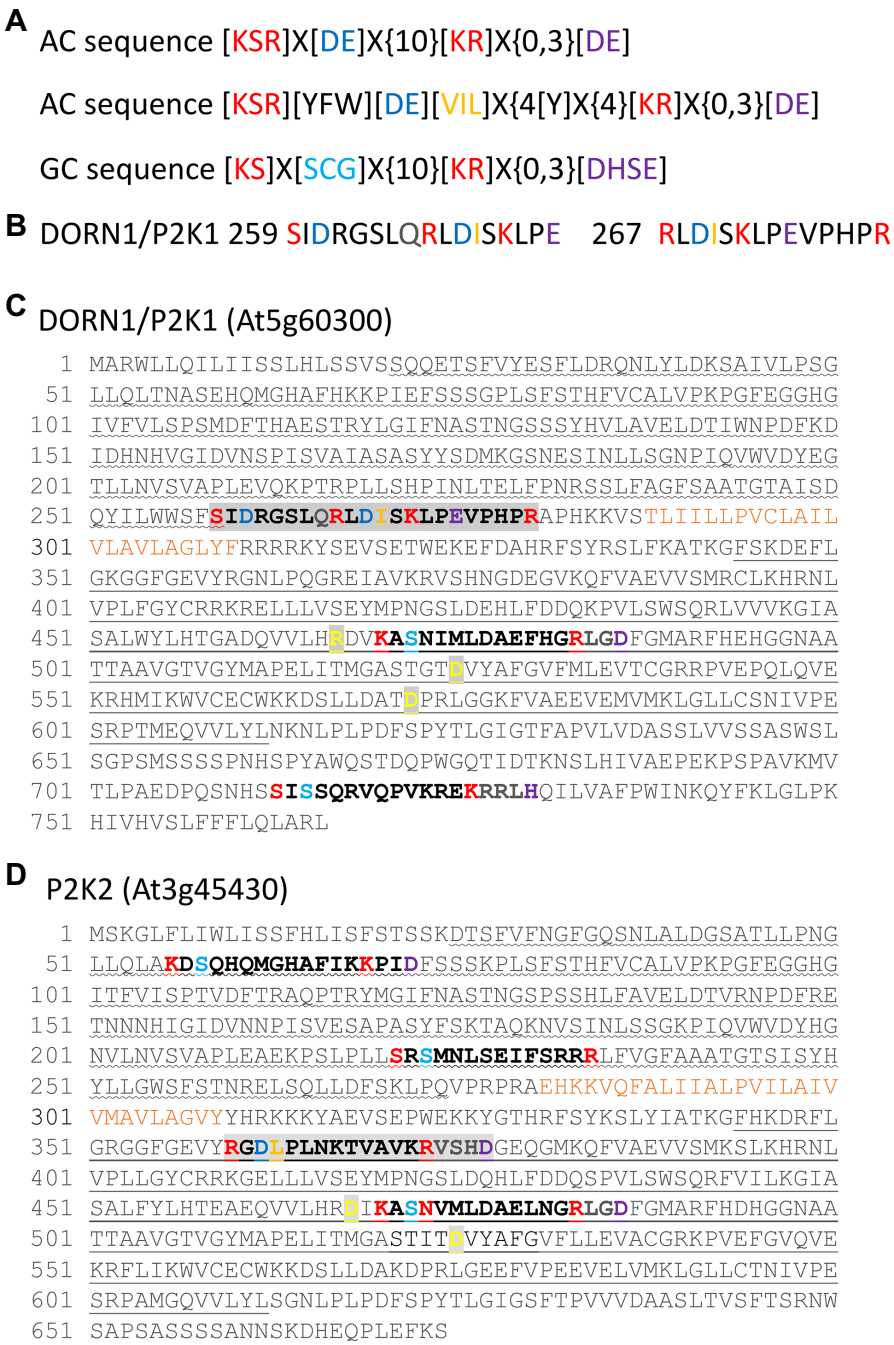
Putative AC and GC sequences in the extracellular ATP (eATP) receptors DORN1/P2K1 and P2K2. **(A)** Top: The ACPred AC prediction tool sequence (Xu et al., 2018b). “X” denotes any amino acid, and “{}” denotes the number of undetermined amino acids. Centre: The AC catalytic center sequence used by Al-Younis et al. (2021). Bottom: The GCPred GC prediction tool sequence (Xu et al., 2018a). **(B)** The two extracellular, overlapping putative AC sequences from DORN1/P2K1 shown separately for clarity. **(C)** Putative AC and GC sequences of DORN1/P2K1. Overlapping AC sequences are highlighted in light gray. The extracellular lectin domain is underlined with a wave, and the intracellular serine–threonine kinase domain is denoted with a straight underline. The transmembrane domain is annotated in ochre. Amino acids found to be important for kinase activity are bold in yellow on a gray background (Choi et al., 2014b; Nguyen et al., 2016; Cho et al., 2017). **(D)** Putative AC and GC sequences of P2K2. The AC sequence is highlighted in light gray. Domains are identified as for **(B)**. Amino acids found to be important for kinase activity are bold in yellow on a gray background (Pham et al., 2020).

To date neither DORN1/P2K1 nor P2K2 has been identified as harboring AC/GC domains. However, the ACPred AC prediction tool sequence [KSR]X[DE]X{10}[KR]X{0,3}[DE] (Xu et al., 2018b; http://gcpred/acpred/; Figure 1A) does reveal possible sequences. Two overlapping sequences are present in DORN1/P2K1 (Figure 1B) that harbor an aspartic acid residue ([D]) at position 3 that should confer ATP-binding specificity, but the sequences are *extracellular*, lying close to the transmembrane domain and beyond the residues already implicated in eATP binding in the lectin domain (Nguyen et al., 2016; Cho et al., 2017; Figure 1C). The first sequence starting at S259 has an aspartic acid residue predicted for cation binding that should follow the 14 amino acid catalytic center motif (Al-Younis et al., 2021) but after an interval of 2 amino acids. The second sequence (starting at R267) lacks a cation-binding residue but overlaps at position 4 with the AC catalytic center motif used recently by Al-Younis et al. (2021; [KSR][YFW][DE][VIL]X{4}[Y]X{4}[KR]X{0,3}[DE]; Figure 1A). Curiously, this second sequence contains the PHPR found previously to be an RGD-binding sequence, relevant to the RGD-containing IPI-O protein secreted by *P. infestans* (Gouget et al., 2006). Potentially, therefore, DORN1/P2K1 has more than one way to bind eATP. P2K2 harbors a putative AC sequence in its cytosolic serine–threonine kinase domain and this sequence conserves an aspartic acid residue for cation binding following the catalytic center motif (Figure 1D). The GCPred GC prediction tool sequence [KS]X[SCG]X{10}[KR]X{0,3}[DHSE] (Xu et al., 2018a; http://gcpred; Figure 1A) revealed two intracellular sequences in DORN1/P2K1, with one harbored in its serine–threonine kinase domain (Figure 1C). For P2K2, two sequences were found in the extracellular lectin domain and one in the intracellular serine–threonine kinase domain (Figure 1D). All of these have the conserved [KS]X[SCG]X{10}[KR] sequence that confers GC function (Wong and Gehring, 2013) and is present in the *Pharbitis nil* GC1 enzyme which has been shown to catalyze cGMP production *in vitro* (Szmidt-Jaworska et al., 2009). The [DHSE] sequence was absent or present after a 2 amino acid gap. The intracellular sequence and proximal extracellular sequence also had limited conservation with the more detailed GC motif [RKS][YFW][CTGH][VIL][FV]G[DNA]X[VIL]X{4}[KR] used to identify AtGC1 (Ludidi and Gehring, 2003; Wong et al., 2018). Both eATP receptors therefore appear to have the requisite sequences to generate cyclic nucleotides, and this now requires experimental validation.

## CNGC2 is Implicated in Cotyledon eATP-Induced [Ca^2+^]_CYT_ Increase

**Figure 2:**
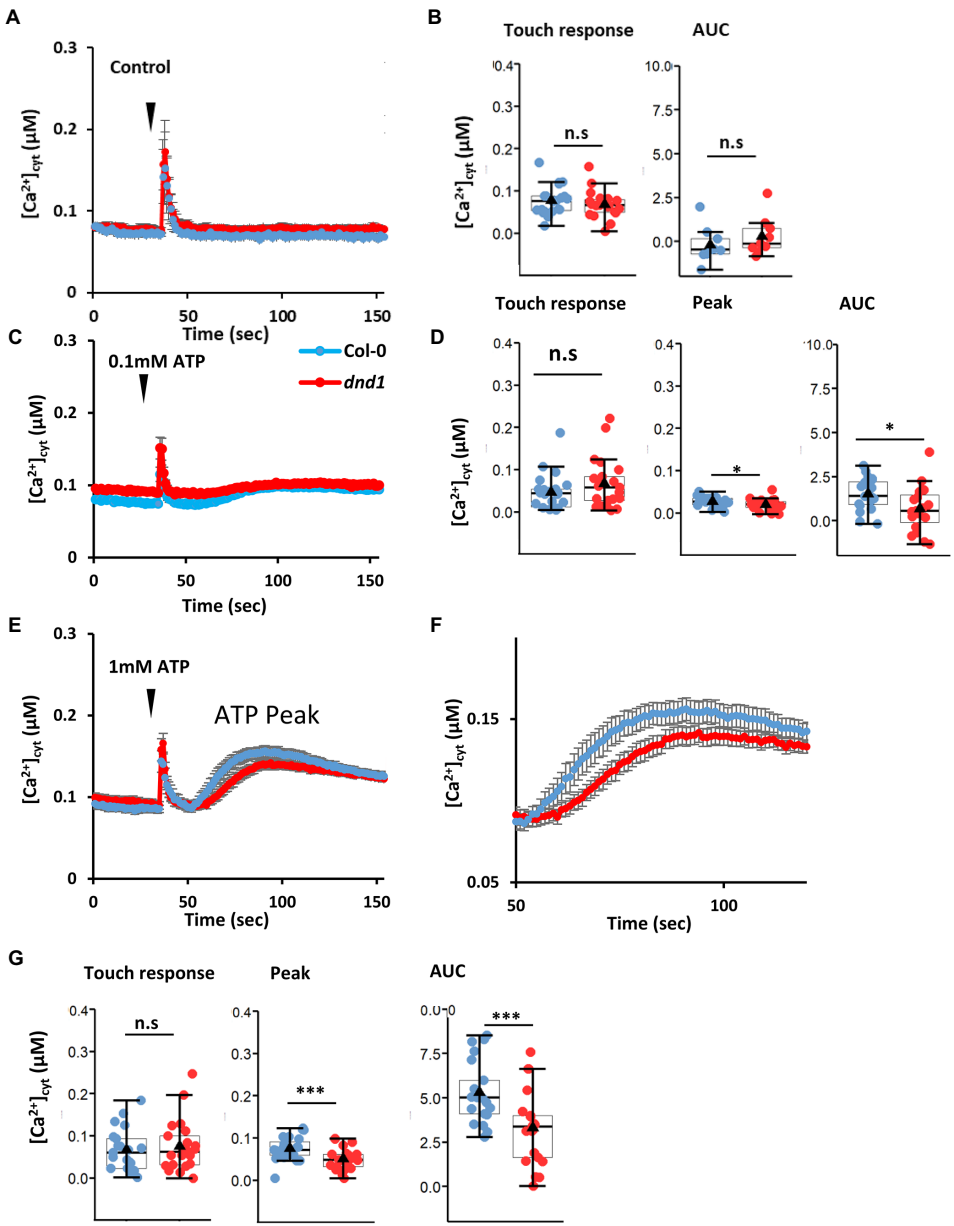
The defense not death 1 (*dnd1*) mutant has an impaired [Ca^2+^]_cyt_ response to eATP. Cotyledons were excised from 14-day-old Col-0 and *dnd1*; cytosolic aequorin was reconstituted with coelenterazine (in 10 mM CaCl_2_, 0.1 mM KCl, 2 mM Tris/MES, pH 5.8) as described by Mohammad-Sidik et al. (2021). Luminescence was recorded from individual samples bathed in 10 mM CaCl_2_, 0.1 mM KCl, 2 mM Tris/MES, pH 5.8 as described by Mohammad-Sidik et al. (2021). As with flg22, ATP causes pronounced acidification of solutions, and so in this study, pH of eATP test solutions was maintained by buffers to avoid acidic pH artefactual responses which can impair interpretation (Westphal et al., 2019). [Ca^2+^]_cyt_ values were calculated as described by Matthus et al. (2019b). **(A)** Mean ± SEM time course of [Ca^2+^]_cyt_ response of Col-0 and *dnd1* to control solution (10 mM CaCl_2_, 0.1 mM KCl, 2 mM Tris/MES, pH 5.8) added at 35s (inverted triangle). **(B)** Touch-induced [Ca^2+^]_cyt_ increase from pre-stimulus baseline and estimated total [Ca^2+^]_cyt_ mobilized [area under the curve (AUC), after baseline subtraction]. **(C)** Mean ± SEM time course of [Ca^2+^]_cyt_ response of Col-0 and *dnd1* to 0.1 mM eATP added at 35s (inverted triangle). **(D)** Touch-induced [Ca^2+^]_cyt_ increase from pre-stimulus baseline, peak eATP-induced [Ca^2+^]_cyt_ increase from baseline, and estimated AUC. **(E)** As **(C)** but with 1 mM eATP. **(F)** Mean ± SEM time course of the eATP response shown in **(E)**. **(G)** As **(D)** but for 1 mM eATP. Results are from 3 independent trials with 9 samples in total per genotype in control trials and 19–21 samples in total per genotype in eATP trials. Each dot in box plots represents an individual recording. The middle line and the triangle in the box plot are the median and mean, respectively. Student’s *t*-test was used for analyzing statistical difference (^***^p < 0.001; ^*^p < 0.05; n.s, not significant).

While there remains a question mark between eATP and cyclic nucleotides, critically there is a precedent of a DAMP-cytosolic cGMP-CNGC2 pathway. Wounding of *Arabidopsis* increases [Ca^2+^]_cyt_ resulting in activation of metacaspase 4. The latter then catalyzes production of plant elicitor peptide1 (Pep1) from its inactive precursor PROPEP1 (Hander et al., 2019). Pep1 can be perceived as a DAMP in a neighboring cell by its cognate plasma membrane receptors PEPR1 and PEPR2. Both of these LRR RLKs are predicted to have cytosolic GC activity, with PEPR1 confirmed experimentally. The cytosolic cGMP activates CNGC2 to effect a [Ca^2+^]_cyt_ increase and downstream defense transcriptional response (Qi et al., 2010). eATP is now reported to act upstream of CNGC2. Wu et al. (2021) reported that eATP (0.1 mM) failed to elicit a plasma membrane Ca^2+^-permeable influx conductance in *Arabidopsis* pollen grain protoplasts of a *cngc2* mutant. This concentration of eATP stimulated pollen germination in the wild type but not *cngc2* (Wu et al., 2021), but it remains unknown whether activation of CNGC2 by eATP would increase [Ca^2+^]_cyt_. Here, the *cngc2* loss of function mutant defense not death 1 (*dnd1*; which constitutively expresses cytosolic (apo)aequorin as a luminescent [Ca^2+^]_cyt_ reporter, Qi et al., 2010) was tested for impaired eATP-induced [Ca^2+^]_cyt_ increase. The *dnd1* mutant of *CNGC2* has a single point mutation causing a stop codon in the third exon that would result in a truncated protein lacking the pore region for ion conduction. *dnd1* has a dwarf rosette phenotype that was also observed here (Supplementary Figure S1). Addition of control solution alone to single, excised cotyledons of Col-0 and *dnd1* only induced the transient “touch response” caused by mechanical disturbance, which did not differ between genotypes (Figures 2A,B). In contrast, addition of 0.1 mM eATP caused a monophasic increase in [Ca^2+^]_cyt_ after the touch response (Figure 2C). A similar pattern was observed in a previous study by Mohammad-Sidik et al. (2021). The response of *dnd1* to 0.1 mM eATP was significantly lower than the Col-0 wild type, both for the maximum [Ca^2+^]_cyt_ increase and estimated total [Ca^2+^]_cyt_ mobilized [estimated as area under the curve (AUC), Figure 2D]. Increasing eATP to 1 mM (Figures 2E,F) also revealed a significant impairment in the response of *dnd1* (Figure 2G) although clearly at both eATP concentrations loss of CNGC2 function allows the majority of the [Ca^2+^]_cyt_ increase to occur. Overall these results show that CNGC2 could be involved in an eATP-induced [Ca^2+^]_cyt_ increase in leaf tissue.

## Discussion

At present, it can only be inferred from existing studies that eATP could increase cAMP or cGMP synthesis to act as second messengers in [Ca^2+^]_cyt_-based DAMP signaling. However, it is now timely to assess experimentally whether eATP does elicit an increase in cyclic nucleotides and whether this is driven by known eATP receptors. Verification of the putative AC/GC sequences in DORN1/P2K1 and P2K2 would mean that phosphorylation of cytosolic targets is not the only way these receptors can relay the eATP signal. Increasing [Ca^2+^]_cyt_ inhibits the kinase activity of the *Arabidopsis* plasma membrane phytosulphokine receptor 1 (PSKR1) and promotes its GC activity (Muleya et al., 2014). It is therefore feasible that the receptors’ initial function is to relay by phosphorylation (which could activate plasma membrane Ca^2+^ channels to increase [Ca^2+^]_cyt_), but increasing [Ca^2+^]_cyt_ switches the receptors’ mode to cyclic nucleotide production. A further layer of regulation could be effected by levels of H_2_O_2_, given the findings of Kuzakova et al. (2020) of dose-dependent stimulation or inhibition of pea (*Pisum sativum*) AC activity. The finding here of putative extracellular AC sequences in DORN1/P2K1 begs several questions. How does the affinity of eATP binding compare to that of the legume lectin domain (K_d_ 46 nM; Choi et al., 2014a)? Under what circumstances would extracellular cAMP be synthesized and to what purpose? Here, it should be noted that plant plasma membrane moonlighting ACs and GCs have low productivity and are held to be within distinct membrane domains containing their target in order to be efficient (Wong et al., 2018; Turek and Irving, 2021). Could the plant plasma membrane harbor a receptor for extracellular cAMP in close proximity to those for eATP? Two decades ago, there were no signs of a receptor for eATP in the *Arabidopsis* genome, but if a measurable output for sensing extracellular cyclic nucleotides could be established, then there is a way forward through forward genetic screens. Similar questions and future directions can also be posed for the extracellular putative cGMP synthesis sites of P2K2.

The finding here that a small but significant component of the eATP-induced [Ca^2+^]_cyt_ elevation requires CNGC2 (at eATP levels consistent with wounding) helps fill a gap in our knowledge on how [Ca^2+^]_cyt_ elevation is achieved and helps indirectly make the case for a role for cAMP or cGMP in DAMP signaling by eATP (summarized in Supplementary Figure S2). However, it remains feasible that CNGC2 could be phosphorylated by DORN1/P2K1 or P2K2 (if downstream of these) to promote Ca^2+^ influx. CNGC2 is already known to act downstream of PEPR1 and PEPR2 in DAMP signaling (Qi et al., 2010) and downstream of flg22 in PAMP signaling (Tian et al., 2019). CNGC2 therefore could be a critical component in coordinating the response to pathogens, which requires both damage and PAMPs to elicit the strongest immunogenic transcriptional outcome (Zhou et al., 2020). CNGC2 has recently been reported to be involved in auxin signaling (Chakraborty et al., 2021) so it could possibly play a part in the restorative growth response of cells surrounding damage sites as these sustain an increase in auxin as a longer-term response (Hoermayer et al., 2020). Much will depend on which cells harbor CNGC2, what its possible sub-unit partners in heterotetrameric channels are to help generate specific [Ca^2+^]_cyt_ signals and their position in the membrane relative to receptors. There are another nineteen CNGCs in *Arabidopsis* that now warrant consideration as components of eATP DAMP signaling, although CNGC14 appears not to be involved in the root’s [Ca^2+^]_cyt_ elevation by eATP (Shih et al., 2015).

## Data Availability Statement

The raw data supporting the conclusions of this article will be made available by the authors, without undue reservation.

## Author Contributions

JS and KW designed and performed the aequorin experiments. YN and LW analyzed the data. LW designed, performed, and analyzed the biomass determinations. JD analyzed sequences, conceived the project, and wrote the manuscript with contributions from the other authors. All authors contributed to the article and approved the submitted version.

## Funding

Funding was from the UKRI BBSRC (BB/K009869/1), the framework of the 3rd call of the ERA-NET for Coordinating Action in Plant Sciences with funding from the BBSRC (BB/S004637/1), the University of Cambridge and Jiangsu University.

## Conflict of Interest

The authors declare that the research was conducted in the absence of any commercial or financial relationships that could be construed as a potential conflict of interest.

## Publisher’s Note

All claims expressed in this article are solely those of the authors and do not necessarily represent those of their affiliated organizations, or those of the publisher, the editors and the reviewers. Any product that may be evaluated in this article, or claim that may be made by its manufacturer, is not guaranteed or endorsed by the publisher.
