# Supplementary material for: Damage Signaling by Extracellular Nucleotides: A Role for Cyclic Nucleotides in Elevating Cytosolic Free Calcium?
Source: Front Plant Sci. 2021 Dec 2;12:788514. doi: 10.3389/fpls.2021.788514 (PMC8675005; doi:10.3389/fpls.2021.788514)
Supplement: Supplementary file 1 [file Data_Sheet_1.PDF]

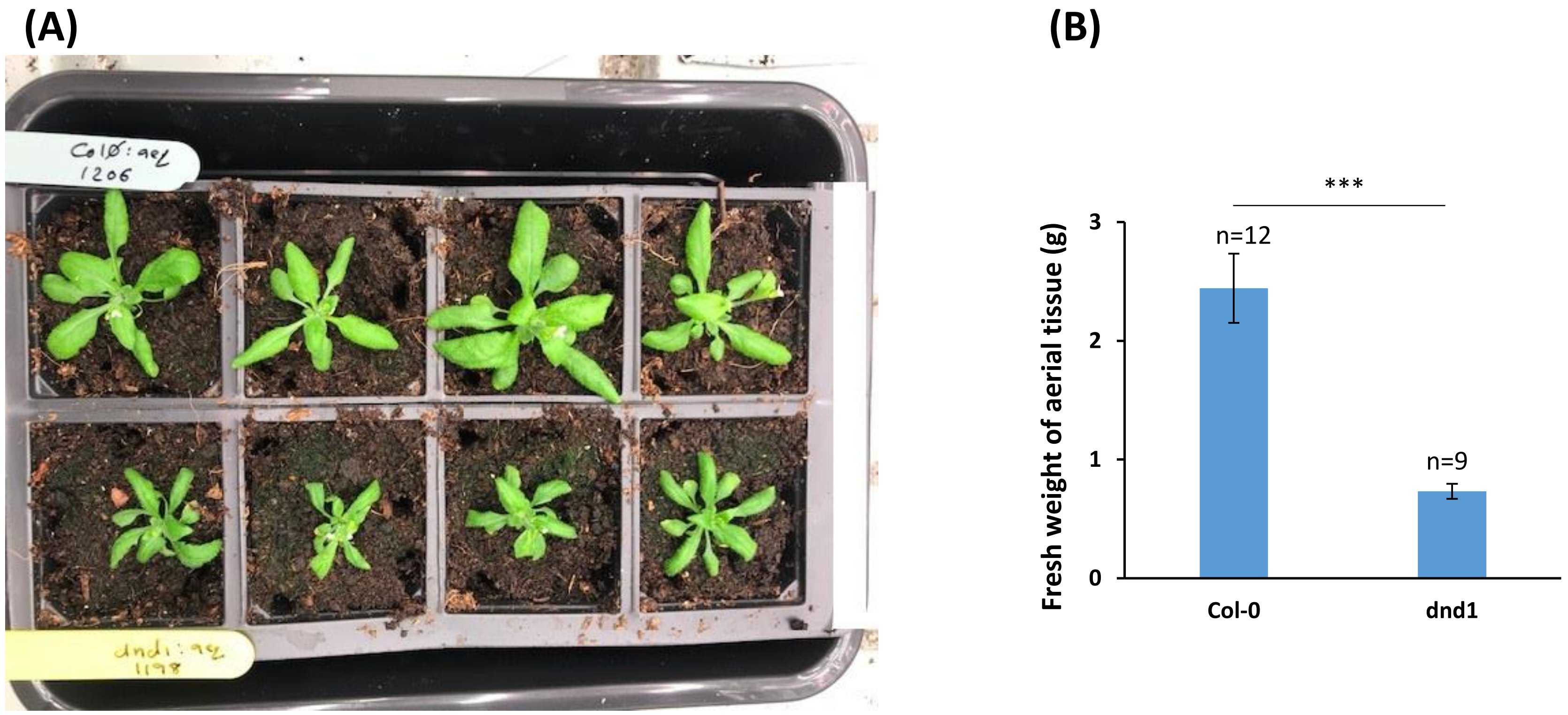

Figure S1. Dwarf phenotype of *dnd1*. A. Col-0 (top row) and *dnd1* (bottom row) grown in soil. B. Mean ± SEM fresh weight of aerial tissue. *n* denotes number of plants. Student's *t*-test was used for analysing statistical difference (***, $p < 0.001$). Plants were grown on Levington's F2 compost for 44 days under long-day conditions (16 h light / 8 h dark) at 200 μmol m^{-2} s^{-1} light intensity, 20°C, 60 % relative humidity.
