# Supplementary material for: Damage Signaling by Extracellular Nucleotides: A Role for Cyclic Nucleotides in Elevating Cytosolic Free Calcium?
Source: Front Plant Sci. 2021 Dec 2;12:788514. doi: 10.3389/fpls.2021.788514 (PMC8675005; doi:10.3389/fpls.2021.788514)
Supplement: Supplementary file 2 [file Data_Sheet_2.PDF]

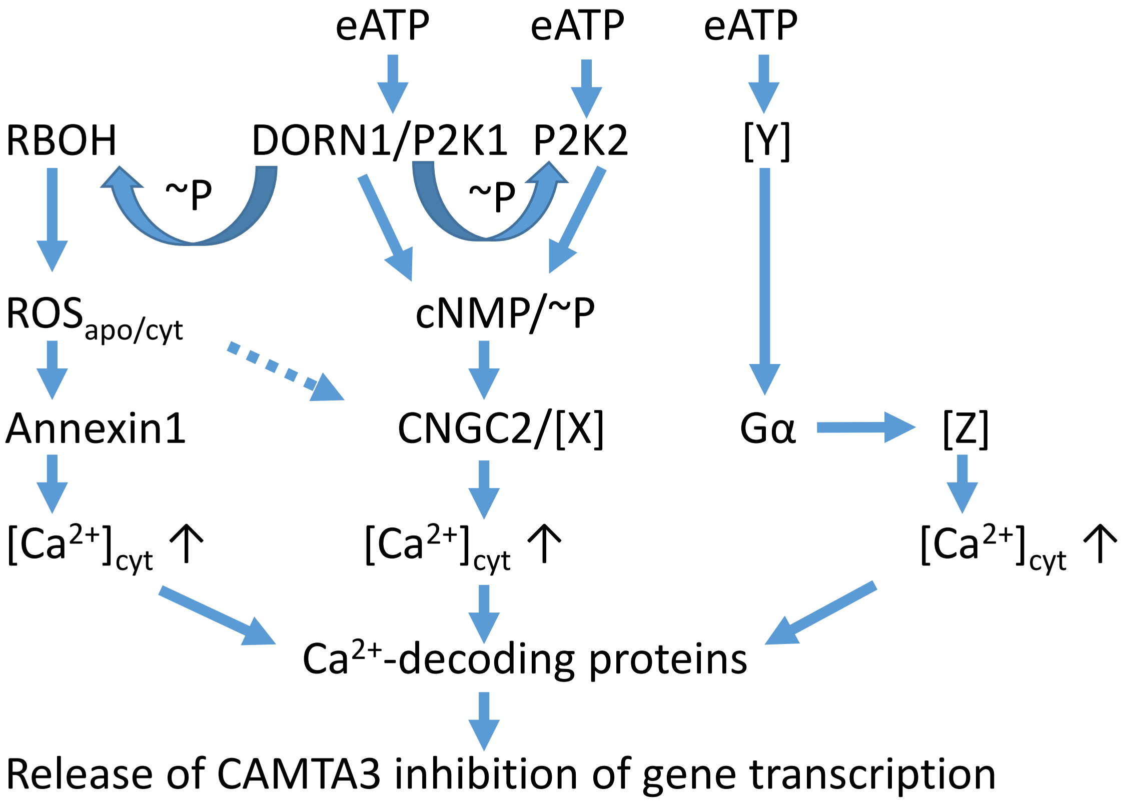

Figure S2. Schematic of the core eATP-$[Ca^{2+}]_{cyt}$ signalling pathway. In *Arabidopsis*, eATP can bind to two known plasma membrane receptors, DORN1/P2K1 and P2K2. DORN1/P2K1 can phosphorylate (~P) P2K2 (Pham *et al*., 2020). DORN1/P2K1 can phosphorylate and hence activate the plasma membrane NADPH oxidase RBOHD in guard cells (Chen *et al*., 2017) buts its RBOH targets in other cells are unknown. As an ROS (Reactive Oxygen Species)-activated plasma membrane Ca^{2+} influx pathway, Annexin1 could lie downstream of RBOHs in roots to elevate $[Ca^{2+}]_{cyt}$ (Mohammad-Sidik *et al*., 2021). Production of cyclic nucleotide monophosphates (cNMP) by the P2K receptors could open CNGC2, working as a plasma membrane homomeric Ca^{2+} channel or as a heteromeric channel working with subunit [X]'s varying with cell type. Opening by P2K-mediated phosphorylation is also feasible. CNGC2 can lie downstream of ROS in flg22 signalling (Tian *et al*., 2019) but it is unknown if that occurs in eATP signalling. Evidence exists for perception of eATP independently of P2K receptors, invoking existence of one or more eATP-binding proteins represented by [Y] (Zhu *et al*., 2017, 2020; Matthus *et al*., 2019a; Smith *et al*., 2021). In roots, this pathway could run through the Gα protein to increase $[Ca^{2+}]_{cyt}$ by opening Ca^{2+} channel(s) represented by [Z] (Zhu *et al*., 2017). In pollen, patch clamp data suggest that the DORN1/P2K1 and Gα converge on CNGC2 (Wu *et al*., 2021). The Ca^{2+} decoding proteins leading to the transcriptional response governed by CAMTA3 (Jewell *et al*., 2019) remain to be determined.
